# Dr. Kinglake on Hydrophobia

**Published:** 1813-01

**Authors:** Robert Kinglake

**Affiliations:** Taunton


*To the Editors of the Medical and Physical Journal.*

GENTLEMEN,

IN your Journal for October last, appeared some observations of mine on Hydrophobia, communicated to you for the purpose of explaining a seeming analogy subsisting between between that dreadful malady and gastritis. It is there held that a striking resemblance obtains between these two diseases in all the essential morbid circumstances that go to constitute both the radical nature and formal character of the two affections.

My object in inviting the public attention to the subject, was to found, on the supposed similitude, a mode of treatment for hydrophobia, that had been ascertained, by my own experience, to be sufficiently efficacious in mitigating the violence and effecting a cure in gastritis. The remedy proposed, was the most extensive depletion of the vascular system by blood-letting, that could be made compatibly with life. To this end, it was suggested that blood should be taken from the hydrophobic patient "to the extent of twenty-four ounces, if deliquium should not be previously induced, repeating it at short intervals as long as either firmness of arterial action or the symptoms of hydrophobic disease might remain."*

In this remedial proposal for a disease that seemed to have baffled every variety of attempt to cure that had been tried, the strong analogy subsisting between the two diseases appeared to me to authorise a similarity in the mode of treatment; and, although a case in point had not occurred to enable me to put the opinion to the test of experiment, it was, in my judgment, better to look to the untried but probable remedy, as a practical resource entitled to confident adoption, than to be running again and again the circle of inefficacious means, that prejudice, reputation, and habit, had regarded as the best, though confessedly unavailing, practice. In this speculation it affords me much gratification to learn the support that it has received from an interesting case recently published in the Bombay Gazette, which I herewith transmit to you for insertion in an early number of your Journal.† In as far as a single instance can go, it does most directly and circumstantially warrant the opinion which I have presumed to submit to public consideration, on the

---

* See Med. and Phys. Jour. for October, p. 276.

† The case of Hydrophobia, alluded to by Dr. Kinglake, was transmitted to us by a correspondent, and printed off before the doctor's communication reached us. We are also indebted to Mr. Shute for a copy of the same case. From the circumstance of three gentlemen, in distant parts of the island, at the same period of time, (for two of the letters bear the same date, and the third is only two days earlier,) forwarding this communication, it must be inferred that Dr. Shoolbred's case has excited considerable interest in this country.—EDITORS.

 highly highly inflammatory nature of the hydrophobic malady, and of its resemblance in this main feature, as well as in the situation and structure of the affected parts, to gastritis.

If the suggested analogy should be adequately verified by farther experience, and an almost unlimited detraction of blood from the vascular system be found in the two cases to be equally efficacious in overcoming the imminent danger attending them, the important fact must be deemed a practical acquisition of inestimable value to the healing art. At all events, during the probationary period of the question, whilst the issue still awaits the result of additional trial, it would seem that enough has been said in the paper referred to on the theory of the subject, and in the subjoined case of the decided utility of applying the principle of that theory to practice, fully to warrant a determined adoption of the same mode of treatment whenever hydrophobia shall actually occur, and to persevere in it as long as its effects shall be curative, or shall even approximate to that desirable influence.

*Taunton,* I am, &c.

*Dec. 5th,* 1812. ROBERT KINGLAKE.

